# Neural Computation via Neural Geometry: A Place Code for Inter-whisker Timing in the Barrel Cortex?

**DOI:** 10.1371/journal.pcbi.1002188

**Published:** 2011-10-13

**Authors:** Stuart P. Wilson, James A. Bednar, Tony J. Prescott, Ben Mitchinson

**Affiliations:** 1 Active Touch Laboratory, Department of Psychology, The University of Sheffield, Sheffield, United Kingdom; 2 Institute for Adaptive and Neural Computation, School of Informatics, The University of Edinburgh, Edinburgh, United Kingdom; Indiana University, United States of America

## Abstract

The place theory proposed by Jeffress (1948) is still the dominant model of how the brain represents the movement of sensory stimuli between sensory receptors. According to the place theory, delays in signalling between neurons, dependent on the distances between them, compensate for time differences in the stimulation of sensory receptors. Hence the location of neurons, activated by the coincident arrival of multiple signals, reports the stimulus movement velocity. Despite its generality, most evidence for the place theory has been provided by studies of the auditory system of auditory specialists like the barn owl, but in the study of mammalian auditory systems the evidence is inconclusive. We ask to what extent the somatosensory systems of tactile specialists like rats and mice use distance dependent delays between neurons to compute the motion of tactile stimuli between the facial whiskers (or ‘vibrissae’). We present a model in which synaptic inputs evoked by whisker deflections arrive at neurons in layer 2/3 (L2/3) somatosensory ‘barrel’ cortex at different times. The timing of synaptic inputs to each neuron depends on its location relative to sources of input in layer 4 (L4) that represent stimulation of each whisker. Constrained by the geometry and timing of projections from L4 to L2/3, the model can account for a range of experimentally measured responses to two-whisker stimuli. Consistent with that data, responses of model neurons located between the barrels to paired stimulation of two whiskers are greater than the sum of the responses to either whisker input alone. The model predicts that for neurons located closer to either barrel these supralinear responses are tuned for longer inter-whisker stimulation intervals, yielding a topographic map for the inter-whisker deflection interval across the surface of L2/3. This map constitutes a neural place code for the relative timing of sensory stimuli.

## Author Summary

To perceive how stimuli move over sensor surfaces like the retina or the fingertips, neurons in the brain must report the relative timing of signals arriving at different locations on the sensor surface. The rat whisker system is ideal for exploring how the brain performs this computation, because the layout of a small number of sensors (whiskers) maps directly onto the layout of corresponding columns of neurons in the sensory cortex. Previous studies have found that neurons located between adjacent cortical columns are most likely to respond when the corresponding adjacent whiskers are stimulated in rapid succession. These results suggest a link between the location of the neuron and the relative timing of sensory signals reported by its activity. We hypothesized that, if the time taken for whisker signals to arrive at a neuron is related to its distance from each cortical column, then neurons closer to a particular column will report stimuli moving towards that particular whisker. In a model approximating the geometry of cortical connections, responses of artificial neurons matched those of real neurons on a wide range of details. These results suggest an important role for neural geometry in neural computation.

## Introduction

A fundamental question in computational neuroscience asks how the brain represents the relative timing of stimuli as they move between sensory receptors, e.g. as a light source moves relative to the retina, or as contact moves between touch sensors on the fingertip. For over 60 years Jeffress’ place theory [1] has remained the dominant model. The idea is that coincidence detector neurons receive input from sensors after delays governed by the distance of the neuron from either sensor. The inter-sensor time difference is encoded by the location of neurons that are active because their connection delays exactly compensate the inter-sensor stimulation interval. The place theory therefore suggests an important role for neural geometry in computing the motion of sensory stimuli.

Strong support for Jeffress’ place theory has been provided by a number of studies of midbrain neurons in auditory specialists like the barn owl, who locate sound sources by resolving small differences in the arrival time of sounds at either ear (see ref. [2] for a review). Evidence from the mammalian auditory system is less conclusive because, for example, rabbit auditory cortex neurons are tuned to inter-ear time differences that are too long to attribute to inter-neuron distances alone [3] (see also refs. [4], [5], and ref. [6] for an alternative mechanism based on slow lateral connections). However few studies have investigated how inter-sensor time-differences might be resolved in specialist mammalian sensory systems.

Tactile specialists like rats, mice, shrews, and seals determine the form and motion of tactile stimuli using prominent arrays of whiskers (vibrissae) on the face [7], [8]. For example, shrews hunting in the dark can use their whiskers to localise particular body-part shapes on fast-moving prey animals [9]. Specific to the whisker system is a precise topographic correspondence between the individual sensor and its neural representation. Deflection of adjacent whiskers A and B on the face evokes the largest amplitude and shortest latency responses in adjacent cortical columns A and B in the somatosensory (barrel) cortex. This precise mapping, as well as observations of sub-millisecond temporal precision throughout [10]–[12], makes the whisker-barrel system ideal for exploring the impact of neural geometry on neural computation.

**Figure 1:**
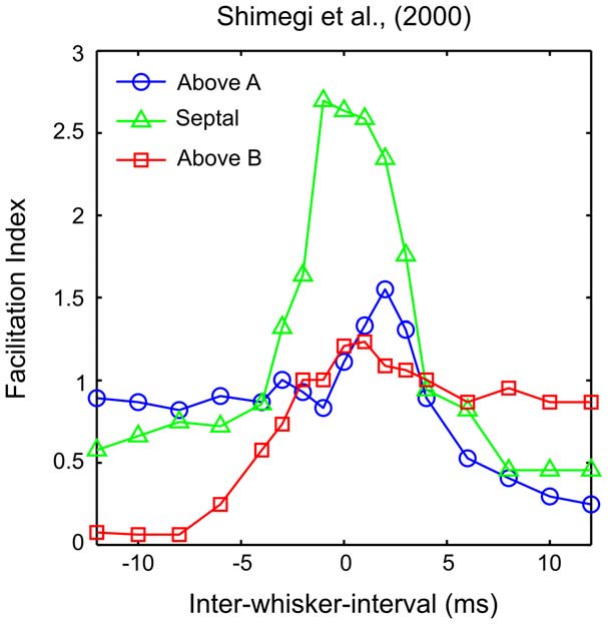
Two-whisker response interactions as reported in ref. [**18**]. L2/3 barrel cortex neurons were grouped by their position relative to the underlying barrel geometry. The spike rate over 50 stimuli at each inter-whisker deflection interval (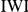) is shown as an average for neurons located above barrel A (blue line, open circles), above barrel B (red, squares) or above the septal region between the barrel columns (green, triangles). 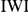 is defined as the time of the whisker A deflection relative to a whisker B deflection at time zero. When the adjacent whisker is deflected after the principal whisker, the response of neurons above the principal barrel is the linear sum of the response to either when deflected independently, as indicated by a facilitation index (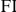) of 1. When the adjacent whisker is deflected prior to the principal whisker, neurons above the principal barrel are strongly suppressed, yielding a 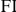 less than 1 and tending to zero for longer intervals. For neurons located between the barrels, longer intervals in either direction yield suppression with 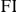 around 0.5. However in these neurons, intervals ranging 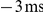 to 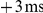 yield responses greater than the sum of the response to either whisker deflected independently and thus 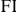 greater than 1. Notice a smaller positive 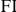 peak in neurons above A when the whisker B deflection precedes by 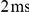. These trends will be used to validate the model. The figure is a visualisation of the data reported in ref. [18], their Figure 8E, obtained from a computer-aided scan; the original error bars and statistical significance indicators are omitted, colour is added, marker styles are changed, and the axes are relabelled for clarity.

A consistent finding across studies in the rat and mouse somatosensory cortex is that responses vary with the time interval between adjacent whisker stimulation [13]–[24]. A useful metric for comparing the response to a two-whisker stimulus to the response to the individual whisker deflection is the facilitation index [17], defined as ‘the response to paired deflection of whiskers A and B divided by the sum of the response to deflection of whisker A deflected alone and the response to whisker B deflected alone’ or 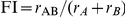. In layer 2/3 barrel cortex (L2/3) in particular, paired stimuli in which the adjacent whisker deflection precedes by 20–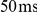 typically evoke sublinear responses (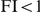). For a range of near-simultaneous deflections, a number of studies have also reported supralinear responses (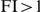), again particularly in L2/3 neurons [16]–[18], [22], [23] (but see ref. [25]). Interestingly Shimegi et al. [18] reported that septa-related neurons in L2/3, located at the midline area between two barrels, were more likely to show response facilitation for short-interval stimuli, whereas barrel-related neurons were more likely to show response suppression by prior deflection of the distal whisker at longer intervals (see Figure 1). Plots of the relationship between the inter-whisker-interval and the response magnitude for individual neurons showed evidence of tuning to particular short intervals. Together these results suggest that the location of the L2/3 neuron relative to the underlying barrel geometry is important in determining its response to a two-whisker stimulus.

One explanation for the different responses of barrel-related and septa-related neurons, as summarised in Table 1, is that they reflect the operation of different mechanisms for integrating adjacent-whisker signals in distinct barrel and septal circuits (see refs. [26]–[28]). However an alternative hypothesis, inspired by the place theory, is that the differences reflect an underlying continuum of responses, which are determined by the location of the neuron with respect to the two cortical columns. This hypothesis would allow for, although it would not require, an essentially homogeneous population in L2/3.

**Table 1:** Relationship between neuron location and paired-whisker response integration.

	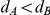	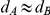	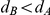
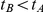	0	0.5	1
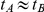	1	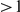	1
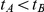	1	0.5	0

Summary of the trends of facilitation index scores (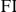), as a function of the relative stimulus timing and neuron location. 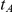 and 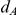 are the deflection time of whisker A and the distance of the neuron from the center of barrel A respectively. Thus the responses are strongly affected by the relative timing of whisker stimuli and the location of the neuron.

According to this alternative hypothesis, the relationship between the inter-whisker deflection interval and the facilitation index in L2/3 neurons may be determined by differences in the arrival times of synaptic inputs that originate from either barrel. These differences may be attributed to inter-soma distance-dependent delays in the feed-forward projection from the major input in layer 4 barrel cortex (L4). This hypothesis is supported by estimates of the speed of the projection between L4 and L2/3 neuron pairs that are relatively slow, around 0.2 meters per second for excitatory and inhibitory post-synaptic neurons [29], [30].

In this paper we show that simulated barrel cortex neurons that receive synaptic inputs with onset times constrained to embody this hypothesis can account for all of the trends relating to the stimulus interval in the data of ref. [18]. We show that a natural prediction of the model is the existence of a topographic mapping of the inter-whisker deflection interval across the surface of L2/3. Specifically, supralinear population responses will peak at short non-zero intervals in neurons located closer to the barrel representing the later of the two deflected whiskers. The responses of individual L2/3 neurons satisfy the basic requirements for a motion detector, and across the population these responses encode a range of stimulus motion velocities. Results therefore suggest that two-whisker timing is represented by a place code in L2/3 barrel cortex.

More generally, the lateral displacement of active neurons due to distance-dependent delays on projections between cortical columns can be used to compute the sequence and timing of events between the sensory stimuli represented by activity in those columns. The results are interpreted as evidence in support of the place theory as a general model of cortical processing of spatiotemporal information.

## Materials and Methods

### The distance-dependent delay hypothesis

We hypothesise that distance-dependent delays associated with inter-columar projections in sensory cortex can be used to extract the relative timing of sensory events. Specifically, delays in the projection from layer 4 (L4) to layer 2/3 (L2/3) barrel cortex might generate selectivity to the inter-whisker deflection interval for adjacent whiskers. To test the hypothesis, the latencies of synaptic inputs to a leaky integrate and fire neuron were constrained to reflect the range of geometries that characterise the L4 to L2/3 projection. To validate the model, we recreated an adjacent-whisker paired-deflection study [18], and compared responses of neurons in different cortical locations to stimuli in which the whiskers were deflected through a range of intervals.

The simplified model is based on three main assumptions, which are described with respect to the validation data in terms of adjacent whiskers A and B, but which in principle apply to a general model of cortical responses to arbitrarily complex multi-whisker deflection patterns.

The first assumption is that, upon whisker stimulation, inputs to L2/3 tend to originate from L4 neurons at the center of the corresponding barrel in L4. Therefore, in the model, the input layer L4 is collapsed down to just two point sources, with activity at each source representing the deflection of the corresponding whisker A or B.

The second assumption is that the excitatory and inhibitory synaptic inputs evoked by deflection of whisker A and by deflection of whisker B arrive at a population of L2/3 neurons situated above and between corresponding barrels A and B. Therefore, in the model, each L2/3 neuron receives just four inputs, although each represents the total contribution of many similar synaptic contacts.

The third assumption is that the time taken for a L2/3 neuron to register a synaptic input is proportional to the straight-line distance between the L4 and L2/3 neuron. Therefore, in the model, we assume that the time of arrival of each synaptic input is a linear function of the distance of the L2/3 neuron from either point source in L4, and we refer to the associated constant of proportionality as the connection speed.

This simplified model of the neural geometry may deviate from the true situation. For example, if the signalling delays are due to the axonal propagation speeds, then delays could be modified by the morphology of L4 axons, which branch vertically and laterally into L2/3 [31], [32]. Delays could also be modified by particular branching patterns that vary systematically with the location of the neuron in the home barrel [33]. We choose not to explicitly model the variety of axonal morphologies, firstly to keep the model formulation simple, secondly because L4 to L2/3 signalling delays are well predicted by the straight-line inter-soma distance [29], [30], [34], and thirdly because post-hoc simulations which considered a laterally-branching axonal morphology did not significantly alter the results. Furthermore, recurrent interactions within L2/3 are not modelled explicitly, because they would occur subsequent to the initial activation of L2/3, and thus could only affect the afferent response after the critical first spike response has been determined (see *Discussion*). Similarly, modelling each L4 input source as a discrete representation of one whisker is justified because multi-whisker responses in L4 are thought to be due to latent contributions from intra-cortical mechanisms [19] (see *Discussion*). The following sections outline how each assumption is represented formally in a model that we refer to as the distance-dependent delay hypothesis. The plausibility of each assumption, the impact of each simplification, and the alternatives to each are considered in *Discussion*.

### A simplified model of feed-forward layer 4 to layer 2/3 connectivity

The thalamocortical volley of excitation from thalamus to L4 and then up into L2/3 [34], [35] is closely followed by a volley of disynaptic inhibition, mediated by a small number of interneurons in L4 [36], with a diverse range of morphologies [32]. We posit that the main excitatory input to L2/3 is derived from direct synaptic connections from excitatory neurons in L4, and the main inhibitory inputs are derived indirectly from excitation of L4 inhibitory interneurons. The circuit therefore consists of three connections: an excitatory connection from L4 to L2/3, an excitatory connection onto the L4 inhibitory interneuron, and an inhibitory connection from the L4 interneuron to the L2/3 neuron.

According to the distance-dependent delay hypothesis each connection has an associated delay. The onset time of the direct excitatory synaptic input at the L2/3 neuron is proportional to its distance from the barrel center. To model the indirect inhibition through an inhibitory interneuron we use a time delay proportional to the L4 to L2/3 distance plus a constant time delay accounting for the distance of the interneuron and its spike generation time.

The circuit therefore has three parameters: the speed of the excitatory pathway between L4 and the L2/3 target neuron (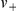), the speed of the inhibitory pathway between L4 and the L2/3 target neuron (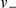), and a fixed latency representing the delayed onset of the spike in the inhibitory interneuron (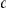) relative to the onset of excitation in L4.

For neurons in the barrel cortex, the principal whisker is typically defined as the one which, upon deflection, elicits the shortest latency and/or the largest-amplitude response. Neurons of a particular barrel column tend to share the same principal whisker, the one which on the face is isomorphic with the position of the barrel in the grid of barrels. For a given neuron all three criteria usually select the same whisker. These constraints can be built into the model if, for progressively longer inter-soma distances, whisker-evoked inhibition arrives progressively earlier than excitation. This pattern of delays requires that inhibitory connections are faster than excitatory connections, and that the onset of inhibition is delayed relative to the excitation. This is achieved in the model by setting 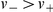 and 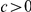.

### Geometry of the L4 to L2/3 projection

**Figure 2:**
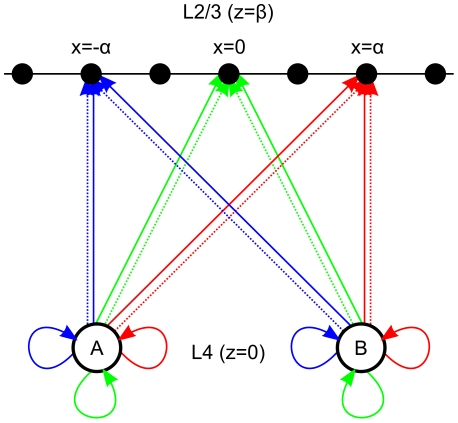
Schematic model of the L4 to L2/3 projection geometry. Input sources A and B are adjacent barrel centers in L4 that respond when corresponding whiskers A or B are deflected. Individual neurons in L2/3 (black dots) receive direct excitatory connections (solid line), or indirect inhibitory projections (dotted line) that are delayed by an additional connection (solid loop). All connections to a neuron above barrel A are shown in blue, those to the midline neuron are shown in green, and those to the neuron directly above barrel B are shown in red.

In the analysis presented by Shimegi et al. [18], against which the model will be validated, L2/3 neurons were characterised by their horizontal location with respect to two underlying barrel columns. The geometry is shown in Figure 2.

In the model axes 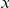 and 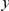 refer to orthogonal axes of the plane tangent to the pia matter of the brain (i.e., the plane tangential to the cortical surface) [37]; specifically 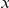 is aligned with barrels that correspond to a row of whiskers on the face, and 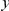 is orthogonal in the ‘tangential plane’. The axis 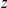 is normal to the tangential plane. Axes 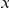 and 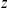 will henceforth be referred to as the horizontal and vertical axes respectively.

In the model, L2/3 neurons will be parameterised only by their horizontal location relative to the two input sources in L4. In effect, this means reducing the three spatial dimensions 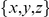 in which intra-cortical connections are defined to just two spatial dimensions 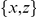 by setting 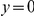. In this way we can define the position of two sources in L4 at 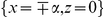. Similarly we can describe L2/3 as a one-dimensional string 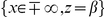 and uniquely describe the location of individual L2/3 neurons along the string in terms of 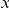. For example the neurons at 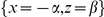,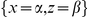, and 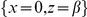 are L2/3 neurons located directly above barrel A, above barrel B, and above the midline respectively.

The Euclidean distance of each L2/3 neuron from the two sources can now be written in terms of 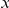:
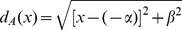
(1)

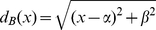
(2)


For the analyses presented in *Results*, the input sources were located at 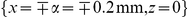 and the two layers were separated by vertical distance 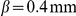. We will henceforth refer to 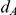 and 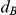 as inter-soma distances.

Reducing the description of the neural geometry in this way makes interpretation of the behaviour of the model tractable, and it allows for a direct comparison with the available electrophysiological data. We note that using an alternative geometry has little impact on the main results, as considered in detail in *Discussion*.

### Incorporating the distance-dependent delay hypothesis into the L4 to L2/3 projection

The L2/3 neuron receives excitatory and inhibitory synaptic inputs from each stimulated whisker. Thus, under two-whisker stimulation, the time of each input is given by:
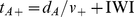
(3)

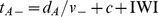
(4)

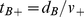
(5)

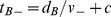
(6)


The inter-whisker interval (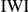) is the time of deflection of whisker A, relative to whisker B, which is always deflected at time 0. Thus if 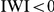 whisker A was deflected before whisker B, if 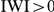 whisker B was deflected before whisker A, and if 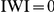 then the whiskers were deflected simultaneously.

**Figure 3:**
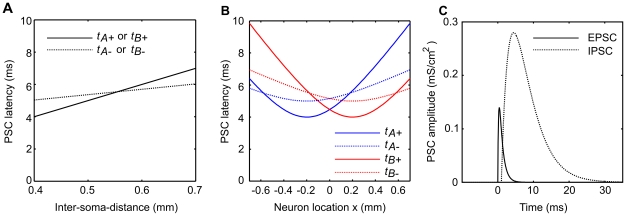
A model of distance-dependent delays in the L4 to L2/3 projection. **A** Distance-dependent delays in the L4 to L2/3 excitatory neuron projection. The onset of the post-synaptic conductance change (PSC) registers at the neuron after delay proportional to distance (minimum 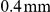), defined by connection speed 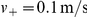 or 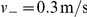 for excitatory (EPSC; solid line) and inhibitory (IPSC; dashed line) pre-synaptic neurons respectively. The inhibitory projection is in turn delayed by a constant temporal offset 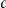. **B** Geometry of the L4 to L2/3 projection. L2/3 neurons are indexed by vertical distance 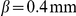 and horizontal location 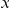, with 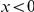 neurons located closer to barrel center A at 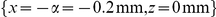, and 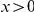 located closer to barrel B at 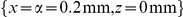. This geometry constrains the PSC onset latencies given by the model in **A** to be hyperbolic functions of 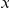. Thus for simultaneous deflections of whiskers A and B the two synaptic inputs arising from deflection of whisker A (blue lines) and B (red lines) arrive in sequence depending on the location of the neuron 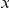. The earliest input arrives at the neuron directly above the barrel center and is excitatory. **C** The timecourse of the excitatory and inhibitory PSC evoked by a whisker A stimulus is shown with relative PSC onset times for neuron 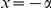.

The relationship between the inter-soma distance and the onset time of excitation and inhibition is illustrated in Figure 3A. The connection speeds were chosen to be 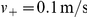 and 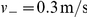, which are in the range of estimates derived from electrophysiological data [29], [30], but we note that similar analyses have estimated speeds as slow as 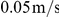 [34]. The constant 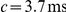 was chosen to delay the onset of inhibition relative to excitation by 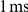 for the neuron located closest to either barrel center, i.e., 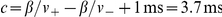.

With the inter-soma distance constrained by the geometry of Equations 1 and 2, the input onset times, described by the linear functions in Figure 3A, become hyperbolic functions of the neuron location 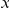, as shown in Figure 3B.

### Leaky integrate and fire model layer 2/3 barrel cortex neuron

The model neuron is a simple integrate and fire neuron with inputs in the form of excitatory and inhibitory post-synaptic conductance changes (EPSCs and IPSCs). Parameters followed those reported by Puccini et al. [38] as a guide for neurons in the barrel cortex.

The time course of each input 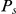, following its onset at time 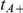, 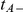, 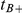 or 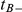, was modelled as a normalised difference of two exponentials:
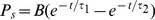
(7)


The normalisation term 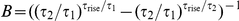, where 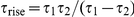, ensures that the potential peaks at 1.

For excitatory synapses 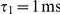 and 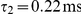 simulating AMPA receptor channel opening [39], and ensuring that excitatory inputs peak at 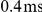. For inhibitory synapses 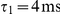 and 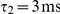 as used by Puccini et al. [38] to model GABA receptor channel opening, peaking later than the EPSC at 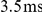 as seen in electrophysiological data (e.g., ref. [40]). The maximum EPSC amplitude was 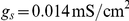 and the maximum IPSC conductance amplitude was 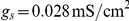 (similar to ref. [38]). The relative amplitude and time course of the excitatory and inhibitory post-synaptic currents are illustrated in Figure 3C.

For the L2/3 neuron we used a standard leaky integrate and fire neuron [41], again with parameters guided by those from ref. [38]:
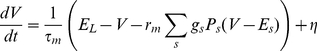
(8)


where the membrane time constant 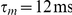, the resting potential 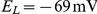, the reversal potential for synapses 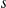 of type inhibitory 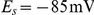, and for excitatory synapses 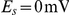. The leak conductance was 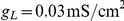 and hence the membrane resistance 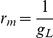. Gaussian noise 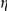 with standard deviation 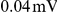 was added to the membrane potential at each time step. Integration was by the forward Euler method (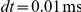).

When the membrane potential reached 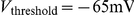 a spike was recorded, and the membrane potential was set to 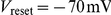.

## Results

The model is validated against the data of Shimegi et al. [18], which show a range of sublinear and supralinear facilitatory responses in neurons in different locations when paired whisker deflections occur at different inter-whisker intervals. In the following sections we show that simulated L2/3 barrel cortex neurons display the same range of interactions observed experimentally when the timing of synaptic inputs is determined by the connection geometry.

### Responses to isolated deflections of the principal and adjacent whisker

To anticipate how a L2/3 neuron might respond to independent deflections of either whisker, we first determine when the onset times of the EPSC and IPSC evoked by deflection of that whisker will be coincident. We derive the time of coincidence by setting the onset times to be equal and rearranging:
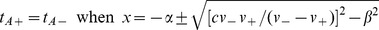
(9)

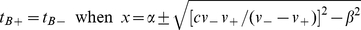
(10)


Therefore we can determine that when 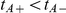 and hence 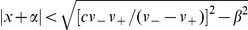 we would expect to see the largest responses to deflection of whisker A because the excitatory input precedes the inhibitory input.

**Figure 4:**
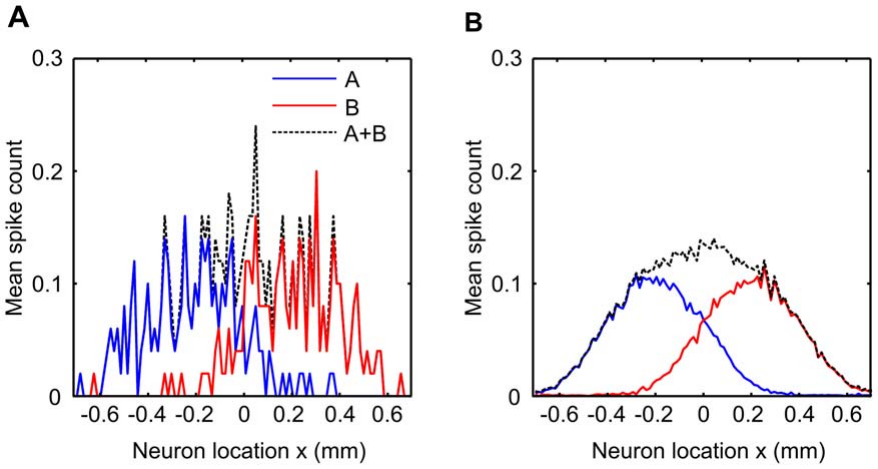
Response to independent deflection of the whiskers. Whisker A (blue line) was deflected 50 times in separate trials, and the average spike count over trials was measured in neurons at different locations in L2/3. Responses are highly variable, but are largest for neurons located directly above barrel A at 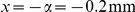 and fall off for neurons further away from the center. Similarly responses to whisker B deflections (red line) fall off with the distance of the neuron from barrel center B at 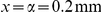. The linear sum of the responses (dashed line) is used later to calculate the facilitation index scores. **B** Responses are shown as means over 5000 stimulus presentations for clarity.

To test this, neurons through the range of 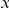 locations were stimulated by applying a deflection to either whisker A or whisker B in isolation. Analogous to the experimental procedure of ref. [18], each trial began 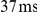 prior to the onset of the first whisker deflection and ended 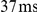 after the onset of the second deflection. Spike counts were calculated over this time window for the results of all simulations, however we note that spikes were precisely timed to the whisker stimuli and so this choice of time window is not critical for the behaviour of the model (see Figure S1). The spike rate is shown as an average over 50 trials in Figure 4A to allow direct comparison with the results of ref. [18], and averaged over 5000 trials for clarity in 4B. As expected, neurons located closer to a particular barrel spike more often in response to deflection of the corresponding whisker. As the distance of the neuron from either source increases, the excitatory and inhibitory inputs evoked by the corresponding neuron register at the neuron closer together in time and thus the window of opportunity in which the EPSC can cause a spike decreases. At longer inter-soma distances, the IPSC precedes the EPSC, and effectively silences the neuron. These observations agree with the notion of the principal whisker as that represented by the barrel closest to the neuron, and which evokes the shortest latency and largest amplitude response.

Figure 4 shows the linear sum of the response to independent deflection of both whiskers. These values for the linear sum are later used to construct facilitation index scores from the average spike counts obtained in paired whisker-deflection trials.

### The timing of synaptic inputs maps between the inter-whisker-interval and neuron location

**Figure 5:**
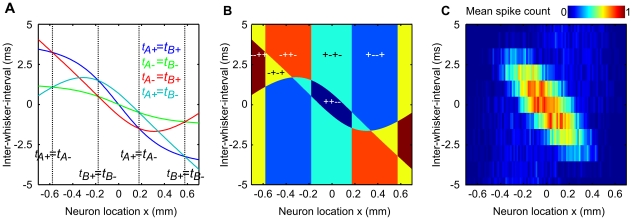
Simulated two-whisker response interactions. **A** Coincident synaptic input onsets. The model equations were rearranged to define the time at which each pair of synaptic input onsets arrives coincidently as a function of the neuron location and inter-whisker interval. **B** These solutions can be used to determine zones in which the excitatory inputs arrive in particular sequence. Neurons close to the midline register both excitatory inputs before both inhibitory inputs when the closer of the two whiskers is deflected after the more distant whisker at short intervals (dark blue zone). Under these conditions we might expect the neuron to display a large response. When these neurons are stimulated at longer intervals (cyan zone) each excitatory input immediately precedes an inhibitory input. As the excitation/inhibition pairs become separated in time the conditions are similar to the independent whisker deflections case and we might expect to observe baseline spiking. For neurons located further from the midline, when the adjacent whisker deflection precedes the principal whisker deflection by longer intervals an inhibitory input precedes both excitatory inputs, and we might expect to see a reduction in the firing rate. **C** Average spike rate measured from simulated L2/3 neurons. Neurons in different 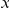 locations were stimulated by paired-whisker deflections through a range of inter-whisker intervals. The colour of each pixel represents the average spike count averaged over 50 trials according to the colour key. The trends in the simulation data confirm the predictions formulated in reference to panel **B**. Neurons located closer to either barrel fired more often in response to a preceding adjacent whisker deflection for a range of short inter-whisker intervals, and showed the weakest responses when this interval was increased. The orientation to the patch of high activity in this space represents a topographic mapping of the two-whisker interval across L2/3.

For independent deflections of either whisker, we have seen that the spike rate is dictated by the sequence and relative timing of the synaptic inputs. Responses to paired whisker deflection stimuli are more complex because they are dictated by four PSCs rather than two and also by the 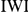. However similar analysis of the relative arrival times of PSCs can be used to anticipate these responses. To this end it is useful to consider regions of the space of possible neuron location and inter-whisker deflection intervals (henceforth 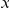
–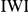 space, see Figure 5A) that are delineated by different ordering of arrival times of the four PSCs.

These regions are delineated by loci representing coincident arrival of each possible pair amongst the four PSCs. Equations 9–10 represent two such pairs. As their solutions are not dependent on the 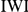, Equations 9–10 describe four loci, which when plotted are straight lines at constant values of 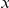 that divide 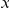
–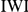 space into five columns in Figure 5A. Solutions for the other four pairs of PSCs can be written as functions of 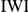 as follows:
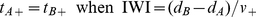
(11)

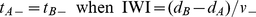
(12)

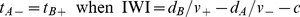
(13)

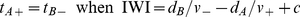
(14)


The solutions to Equations 11–14 are also plotted in Figure 5A, and they further divide the columns into ‘rows’.

For each region of the graph we can use the equations to state the sequence of inputs for each synaptic pair. This is done by setting all 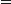 signs to 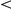 signs in Equations 9–14. The eight inequalities that define each region of the graph can then be combined to give the order of all four synaptic PSCs, and the twenty-four possible PSC orderings take the form 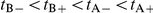, for example, in the top-left region of 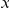
–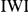 space shown in Figure 5A.

Considering now only whether each synaptic event in the input sequence is excitatory or inhibitory, we can describe the input to the L2/3 neuron more simply. This effectively reduces the twenty-four PSC sequences to just six different orders in which excitation and inhibition can arrive at the neuron. Figure 5B shows how each of the six orderings delineates a zone in 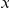
–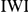 space.

For a range of short interval stimuli, neurons situated near the midline receive both excitatory inputs before both inhibitory inputs. They receive inputs in the order 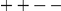, which can be read as ‘two excitations followed by two inhibitions’. This zone is coloured dark blue in Figure 5B. It is in this zone that we would expect to observe the greatest spike rate because neither IPSC precedes the EPSCs. Notice that this zone is oriented diagonally in 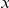
–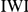 space, and therefore neurons in different locations near the midline will prefer a range of (short) 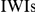.

Similarly we can expect that the greatest suppressive interactions will be displayed in the yellow (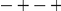), brown (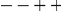), and orange zones (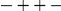), in which an IPSC event is always registered first. Of these zones the orange will be expected to yield the smallest suppression as the second IPSC is preceded by both EPSCs.

In the blue zone (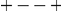) we might expect just one of the whisker deflections to evoke a response, as the second EPSC will be silenced by two preceding IPSCs. In the cyan zones (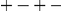) both EPSCs are followed immediately by an IPSC. Therefore we might expect that if the two EPSC/IPSC pairs are separated sufficiently in time for the neuron to respond to them independently, i.e., if the first inhibition has little effect on the second excitation, then the response will resemble the linear sum of that evoked by either whisker deflected independently, and hence the facilitation index score here will be around one.

### Responses to paired whisker deflections encode short inter-whisker intervals

Neurons through the range of 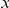 locations were stimulated by applying paired deflections to whisker A and whisker B in sequence. By analogy with the experimental procedure of ref. [18], each trial began 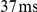 prior to the onset of the first whisker deflection and ended 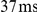 after the onset of the second. The spike rate is shown as an average over 50 trials in Figure 5C.

As anticipated, the greatest activity was evoked in neurons around the midline (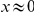) when the whiskers were deflected through a range of short inter-whisker intervals (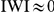). Within this range neurons located left of the midline and therefore closer to barrel A responded maximally to slightly positive inter-whisker intervals where whisker B was deflected before whisker A. Neurons to the right of the midline and therefore closer to barrel B responded maximally when whisker A was deflected before whisker B at short intervals.

For intervals longer than around 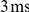 in either direction, and for neurons further from the midline than around half a millimetre, responses were much smaller. In a region of 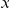
–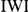 space roughly corresponding with the light blue zone in Figure 5B, responses were more variable at around 0.2 spikes per stimulus.

These results from the full spiking model fit well those expected based on the relative timing of the synaptic inputs. Thus changing the relative timing of the synaptic inputs with distance-dependent delays alters the response of the neuron to paired whisker stimuli in a predictable way. A major feature predicted by the simulation data is a mapping of short interval stimuli to the location of the most active L2/3 neuron.

### Inter-whisker interval tuning in individual L2/3 neurons

The simulation data presented thus far suggest that distance-dependent delays in the L4 to L2/3 projection can generate a spatial encoding of the relative timing of whisker inputs for short interval stimuli. But to what extent do these observations match up with experimental data? To answer this question we look first at the responses of individual model neurons to the range of different interval stimuli.

**Figure 6:**
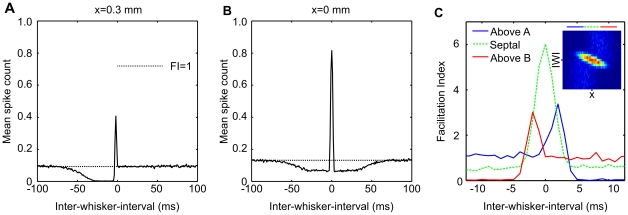
Comparing the neural and simulated data. **A** Mean spike response for the individual neuron 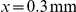 stimulated through the range of inter-whisker intervals. Here we show the spike rate as an average over 5000 trials for clarity. The sum of the average response to either whisker deflected independently is shown by the dotted line. At positive intervals, when the principal whisker B is deflected first, responses fluctuate around baseline, whereas for longer negative 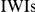 the response is suppressed before recovering at intervals upwards of 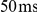. The peak response for this neuron is at 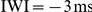. **B** Equivalent data for a neuron at the midline (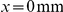). Responses to single and multi-whisker stimuli are symmetrical with respect to the inter-whisker-interval. Responses are suppressed to around 50% of the baseline firing rate for longer 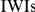 in either direction but are recovered for 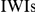 larger than 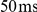. Peak responses are evoked by simultaneous whisker deflections. These plots are similar to those for individual L2/3 neurons reported in refs. [17], [18]. **C** Average response interaction for neurons located above or between the barrels. The data in Figure 5C are reproduced in the inset (for 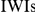 ranging 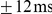) and are shown as means over neuron location in the main plot. Means were taken with respect to groups of neurons ‘above A’ (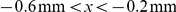), ‘above B’ (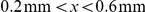), and ‘septal’ between the two (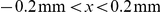). The divisions are depicted by the position and length of the coloured bars above the inset. This plot should be compared directly with the electrophysiological data presented in Figure 1. Each of the major trends are reproduced by the model, including the secondary smaller peak in the above A data. In addition the model data contains a peak in the above B data, which is not clearly present in the experimental data.

Figures 6A and 6B show the average spike rate for an individual neuron located either close to barrel B or between barrels A and B respectively. The neuron in Figure 6A was located approximately 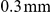 to the right of the midline. Also indicated in the figure is the linear sum of the response of this neuron to either whisker deflected in isolation. Where paired stimuli evoke responses equal to this value, a facilitation index of 1 would be measured and we would conclude that no facilitatory interaction had occurred. Where it is less, suppression would have been measured, and where it is greater facilitation would have been measured. The neuron in Figure 6A shows no facilitatory interaction when whisker B (the principal whisker) is deflected prior to the adjacent whisker A. However for slightly negative intervals strong facilitation was measured, with the average spike count exceeding the linear sum baseline three-fold or more around a peak when whisker A is deflected 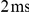 before whisker B. When whisker A precedes by more than 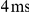 the response is strongly suppressed and almost no spikes are evoked. The suppression recovers towards the linear sum baseline for intervals exceeding 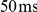.

For the example midline neuron shown in Figure 6B facilitation appears more symmetrical around the zero inter-whisker interval. Facilitation peaks for simultaneous intervals and fluctuates around baseline for longer intervals in either direction. The peak in the average spike count is larger than that for the previous neuron, as is the linear sum response used to compute the strength of its facilitatory interaction.

Equivalent plots for individual L2/3 neurons, found in refs. [17], [18], [23], [24], display similar qualitative trends to those in Figure 6A and 6B, in terms of both the facilitatory interactions and of the average spike counts for independent and paired whisker stimuli.

### Interval tuning over the population is a good match to the experimental data

In Figure 6C we group the L2/3 neurons by location as either above barrel A, above barrel B or in the septal region between the barrels. This allows for a direct comparison between the simulation data (Figure 6C) and the available experimental data of ref. [18] (compare with Figure 1).

The simulation data share many of the qualities of the experimental data, as summarised in Table 1. Septal neurons show a large facilitatory peak for near simultaneous paired whisker deflections and for longer intervals in either direction respond with an average 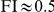, equivalent to the response to either independently deflected whisker. Neurons located above barrel B display on average a lesser facilitatory peak at 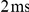 interval stimuli, are suppressed by prior deflection of whisker B, and display no facilitatory interactions when whisker B is deflected first.

Geometry in the model is symmetrical about the midline and therefore the responses are symmetrical about the zero inter-whisker interval. Therefore the above barrel B population display the exact opposite interactions with respect to the interval compared with the above barrel A population. This includes a lesser peak for 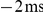 interval stimuli not apparent in the electrophysiological data. Notice too that the peak of the septal group in the experimental data is for a slightly negative inter-whisker interval. We will shortly demonstrate how an extension to the model, which introduces asymmetries related to the direction in which each whisker is deflected, may account for these differences. For now we note that the population response predicted by the model affords a good match to the experimental data.

### A place code for the inter-whisker deflection interval across the surface of L2/3

Instead of asking how L2/3 neurons in particular locations respond to different interval stimuli, we can ask how particular interval stimuli are represented across the population of L2/3. It is particularly important to consider the population response because even the most effective stimuli typically elicit less than one spike per stimulus in any particular neuron, and so individual spikes yield ambiguous information about the stimulus [42].

**Figure 7:**
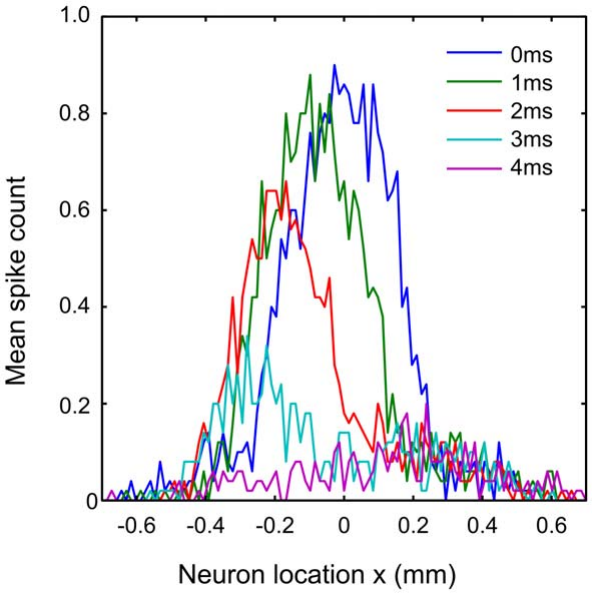
Predicted population place code for two-whisker timing. The mean spike rate plotted against neuron location reveals the population response to various inter-whisker interval stimuli. The peak response decreases and is shifted across the horizontal extent of L2/3 by stimuli varying in interval from 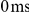 to 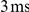 interval. Thus distance-dependent delays in the projection from L4 to L2/3 barrel cortex, coupled with the geometry of the projection, represent a mechanism by which the relative timing of two-whisker stimuli can be encoded by the population activity in L2/3 barrel cortex, for inter-whisker intervals ranging 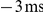 to 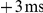.

Figure 7 shows the distribution of average responses across the population for a range of positive intervals. Each of the short inter-whisker deflection intervals is clearly associated with a tuning curve across the population, with a peak that shifts to the left (negative 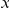) and scales systematically with the increase in interval. Negative intervals also evoke symmetrical results, i.e., a shift in peak responses towards neurons on the right, but we do not show them in the figure for clarity.

Viewed in this way, it is clear that the model predicts the existence of a topographic map for the inter-whisker deflection interval across the surface of L2/3 barrel cortex. According to the model, paired whisker stimuli should elicit supralinear responses and display a systematic shift in tuning across the population for stimulus intervals ranging 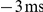 to 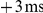.

As well as the representation of the inter-whisker interval across cortical space, it is useful to consider how the stimulus is represented in the timing of spikes. Inspection of maps for the spike timing revealed that in paired-whisker stimulations, spikes were precisely timed to the whisker stimuli. Moreover the largest responses reflected a combination of the delayed response to the principal whisker, as well as the superposition of excitatory influences from both whiskers (see Figure S1). Therefore the model predicts that the effects measured by ref. [18] primarily operate on the first somatosensory-evoked spikes in L2/3.

### Introducing response asymmetry via deflection direction

Barrel cortex neurons are selective for the direction in which the whiskers are deflected. The mechanism thought to underlie directional selectivity in L4 neurons is similar to that which we have outlined for two-whisker timing, but with distances measured in degrees from the preferred stimulus direction [38], [40]. Several studies have suggested that direction preferences vary systematically within the barrel column, such that deflection of the principal whisker to the left or right is correlated with increased activity in neurons located to the equivalent left or right of the barrel column [43], [44]. Therefore we can model the effect of deflecting the whisker in either direction by moving the L4 point source for that whisker in either direction in L4.

**Figure 8:**
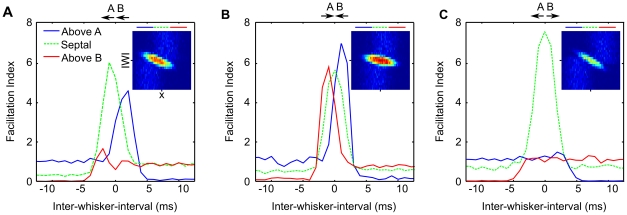
Direction-specific interactions. There is evidence that leftward or rightward deflections of the principal whisker tend to excite L4 neurons situated on the left or the right of the barrel respectively. Therefore to simulate the expected effect of deflecting the whiskers in different directions, we offset the center of activity in the model L4 by 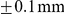. **A** As in ref. [18] both whiskers were deflected to the left, as indicated by the pairs of arrows above each plot. The relationship between inter-whisker-interval and neuron location is the same but shifted for increasing intervals to neurons closer to barrel A. The resulting asymmetry is of the same form as that in Figure 1, increasing the secondary peak in above A neurons, decreasing that in the above B group, and shifting the septal group interval tuning negatively. **B** If the whiskers are deflected toward each other, intra-cortical distances are effectively shortened and the model predicts that facilitatory interactions will be distributed more evenly across L2/3. **C** Conversely if the whiskers are deflected away from each other, distances are increased and all facilitatory interactions are confined to the septal region. The conditions represented in panels **B** and **C** have not yet been conducted experimentally and could therefore be used to falsify the model.

Accordingly, to represent a deflection of whisker A to the left (away from whisker B) we offset the point source in L4 that corresponds to whisker A by a fixed distance 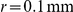 to obtain a new source location at 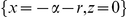. Deflecting whisker A to the right means moving the point source to 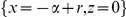 and similarly deflecting whisker B to the left or right means moving the second source to 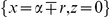. For two whiskers and two deflection directions, possible combinations are both deflections to the left (leftwards), both right (rightwards), A left & B right (outwards), and A right & B left (inwards). Results obtained from the model in these conditions are summarised in Figure 8.

For the analysis shown in Figure 1, Shimegi et al. [18] deflected both whiskers to the left, and so we consider the leftwards condition first (Figure 8A). Conditions leftwards and rightwards produce symmetrical effects and so we only show results for the former. In the leftwards condition, the relative projections, distances, and geometry are identical to the case where the stimulus originates from the barrel centers. However, each projection is shifted to the left, and so each neuron inherits the input timing of that located 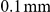 to the right. As a result the effects are still symmetrical but they are symmetrical about a new midline that is shifted to the right at 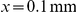. When we average the data across groups defined in terms of the original midline at 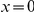, as in Figure 8A, we observe systematic asymmetries in the results. The facilitatory peak in the above A group is increased, that in the septal group is shifted towards negative inter-whisker intervals, and the peak in the above B group is decreased. Thus by introducing a topology associated with the stimulus deflection direction, the model can account for each of the previously unexplained observations in the original data.

This account is also consistent with the observations of Shimegi et al. [18] and Kida et al. [23] (but not ref. [45]), that preferences for the deflection direction of the principal whisker are strongly correlated with those for the adjacent whisker deflection direction, and with the deflection direction evoking facilitatory interactions when both are deflected in that same direction at short intervals.

Predictions of the model for the two stimulus conditions not yet tested experimentally, inwards and outwards, are shown in Figure 8B and Figure 8C. Deflected towards one another (Figure 8B), as may occur when the whiskers encounter a concave stimulus shape, the two stimuli should be represented in the two adjacent sides of the corresponding barrels. This configuration effectively shortens all connection distances, and expands the zone in which both excitatory inputs precede both inhibitory inputs across 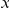. Thus the facilitatory interactions are distributed more broadly across the population, and we would expect to see more similar facilitatory peaks amongst the three neuron groups. Conversely if the two whiskers are deflected away from one another (Figure 8C), as may occur when the whiskers encounter a convex stimulus shape or during divergent whisking movements [46], inputs originate from distal sides of the barrels. This configuration squeezes the zone in which we expect to see facilitatory interactions with respect to 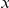, and concentrates them under a single peak in the septal neuron group. Demonstration of effects to the contrary could be used to falsify this aspect of the model.

### An approximate non-linear neuron model reproduces the facilitatory interactions

The particular neuron model from which the previous results have been derived was chosen to allow comparison of the results with real biological neuron data. We have shown how the sequence of synaptic inputs due to distance-dependent delays can change the output of the neuron, but we have not yet determined the origin of the non-linear effects underlying the observed facilitatory interactions. To understand this better we tried to reproduce the effects using as simple a neuron model as possible.

We found that all of the trends in the full model simulations could be reproduced using a simple linear filter neuron model. The reduced model is:
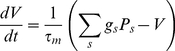
(15)


where 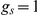 or 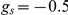, with output squashed using the logistic output function:
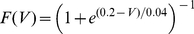
(16)


The range of facilitatory interactions can be seen if we interpret either the maximum or the mean value of 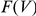 over time as the spiking probability for each stimulus trial.

The logistic output function performs the role of the thresholding operation in the full model. Its form in the full model is affected primarily by the noise, which has a similar effect to the slope of the sigmoid (slope parameter  =  0.04), and the relationship between the firing threshold and the synaptic weights and reversal potentials, which essentially sets the inflection point of the sigmoid (inflection point  =  0.2).

Because both neuron models yield comparable stimulus-evoked interactions, we can be confident that the thresholding non-linearity in the full neuron model, as approximated by the sigmoidal output function in the simpler neuron model, can account for the observed non-linear effects. Comparing the two models in terms of the spike probability is valid in this instance because we observed that in the full model neurons generate less than one spike per stimulus.

## Discussion

We have demonstrated how a model of the geometry of projections within the barrel cortex can generate a range of responses to paired whisker-deflection stimuli that are similar to responses measured in rat L2/3 by Shimegi et al. [18]. The main finding is that distance-dependent delays on projections from L4 to L2/3 can affect how inputs from adjacent whiskers are integrated by a non-linear neuron, in a way that is dictated by the location of the neuron relative to the underlying columnar structure. The data against which the model was validated [18] suggest that neurons located between the barrels combine whisker inputs supralinearly through a small range of inter-whisker deflection intervals (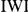), and when the principal whisker deflection is preceded by deflection of the adjacent whisker at longer 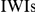 the inputs are combined sublinearly. In the model a discrepancy between the arrival times of excitatory and inhibitory inputs can account for each of the observed trends in the available electrophysiological data. According to our hypothesis, this discrepancy is governed by the lateral displacement between the input neurons and their targets. Therefore the discrepancy is a continuous function of the location of the neuron, and hence the range of non-linear responses is mapped continuously across the surface of L2/3. As a consequence, the model predicts that a range of short 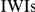 are mapped continuously across a zone of supragranular barrel cortex located between the barrel centers. This mapping constitutes a place code for the timing of the two-whisker stimulus, wherein the stimulus motion velocity (i.e., the 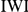) systematically shifts the location of neurons that spike with the greatest probability.

It is useful to consider these findings in the context of the more general problem of encoding sensory stimulus motion. According to ref. [47], the general requirements for a motion velocity detector are threefold. First, two samples or more are required to specify a motion vector, so the detector must receive two or more input signals. Second, the inputs must be asymmetrically processed, such that swapping two inputs registers a change in the output. Third, the inputs must be combined in a non-linear fashion in order that the response to stimuli in different directions is not equal to the mean response over all directions. Our results suggest that responses of individual L2/3 barrel cortex neurons satisfy each of these conditions. Inputs arising from adjacent whiskers and originating from foci in adjacent cortical columns are asymmetrically delayed in their projection to supragranular cortex. The inputs are integrated by individual L2/3 neurons by the non-linear processes involved in spike generation. Hence we propose that one function of the the L4 to L2/3 projection is to encode the stimulus motion velocity defined in terms of inter-whisker contact times.

### Simplifications and assumptions of the model

A major simplification we made in order to construct the model was to explicitly simulate only four synaptic contacts per neuron, whereas real L2/3 neurons receive hundreds of synaptic contacts originating from L4 [48]. Where possible, the parameters of the full neuron model were derived from existing models or electrophysiological data. However to compensate for the decrease in afferent drive, the spiking threshold was lowered from a realistic 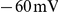 to a low 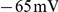. In the final results section we showed that the behaviour of the model is not sensitive to the form of the neuron model chosen, but that each of the trends in the electrophysiological data can be reproduced using a simple sigmoid output function neuron, as used in previous models of the barrel cortex [49], [50].

Another simplification was to relate the delay on each projection to the straight-line distance between the input and its target. This choice was motivated by several studies reporting an approximately linear relationship between the straight-line inter-soma distance and the associated delay [29], [30], [34]. However, the axons of L4 neurons tend to project vertically into L2/3 before turning to branch laterally [51]. Therefore it may be appropriate to consider the Manhattan distance, the vertical plus the horizontal distance, defined in the model by rewriting Equations 1–2 to be of the form 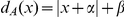. This change has the effect of changing the hyperbolic relationship between 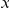 and the synaptic onset latency into a piecewise linear relationship. Each of the zones of synaptic input sequence is maintained in 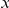
–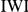 space; hence using the Manhattan distance to compute synaptic input latencies does not change the form of the main results when they are recalculated using this alternative geometry.

The model relies implicitly on the assumption that connections between L4 and L2/3 are organised on a finer spatial scale than that defined by the column boundaries, such that the location of the L2/3 neuron determines its response properties. Evidence from several studies supports this assumption. For example calcium transients measured between pairs of neighbouring L2/3 neurons located above the barrel centers are more highly correlated than those between pairs of distant neurons located above the barrel borders [52]. These data suggest that L2/3 neurons receive input from particular regions of the L4 barrel according to their tangential location in the column [52]. More evidence for a sub-columnar spatial resolution of connections is provided by a correlation between the maximally effective direction of whisker deflection for L4 and L2/3 neuron pairs in vertically aligned sub-regions of the barrel column [43]. Similarly, connected thalamic and L4 neuron pairs share tuning to the whisker deflection direction [53].

The mechanism by which the model accounts for tuning to inter-whisker interval is essentially the same as that thought to underlie tuning for the deflection direction in L4 [38], [40], [54], [55]. In both cases the relative latency of inhibition creates a short ‘window of opportunity’ in the post-synaptic neuron, in which excitatory input representing the preferred stimulus can evoke a response. The dependency of the preferred inter-whisker interval on the connection geometry raises the intriguing possibility that tuning for deflection direction in L4 is inherited from the geometry of the thalamo-cortical projection. A reported topographic organisation of directional preferences about the barrel center in L4 could be inherited from a map of direction preferences measured along the major anatomical axis of the thalamic input barreloid [56]. This idea seems plausible given that thalamocortical axon conduction times range from 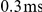 to 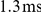 [57], and that latencies ranging 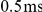 to 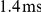 can account for responses to preferred and anti-preferred stimuli respectively [38], [40].

To account for the data of ref. [18], the model requires that at short inter-soma distances excitation precedes inhibition and for longer distances inhibition precedes excitation (see Figure S2). This we attributed to differences in axonal conduction velocity on excitatory and inhibitory projections into L2/3. The origin of the faster inhibition is unlikely to be mediated by L2/3 interneurons, because excitatory connection speeds from L4 to L2/3 interneurons are similar to those from L4 to L2/3 excitatory targets (compare ref. [30] and ref. [29] respectively). The origin is also unlikely to be thalamocortical, because L4 interneurons and L4 excitatory targets are excited after comparable latencies [58], although interneurons are excited via slightly thicker, shorter, and thus faster thalamocortical axons [59]. Therefore we suggest that differences in speed may be attributable to morphological differences between the axons of L4 inhibitory and L4 excitatory neurons; L4 interneurons are known to branch into L2/3 and extend well beyond the boundary of the vertically aligned barrel [32]. To our knowledge, the axonal conduction velocities for this connection have not been directly measured. Therefore the critical quantitative prediction, that the L4 inhibitory axonal conduction speed must be faster than the L4 excitatory speed, can be used to validate the model in a future experiment.

Because each input source in L4 represented the deflection of one whisker, the present model assumed no contribution of sub-cortical mechanisms to the integration of multi-whisker signals. To a first approximation, the barrels in L4 can be considered as functionally separate processing units [34], [37]. Moreover, although non-linear multi-whisker responses can be evoked in L4 neurons [14], [15], [60], much of the effect may be due to intra-cortical rather than thalamocortical mechanisms [19], which are most pronounced in non-granular layers [24], [61], and which would shape responses only after the first stimulus-evoked spikes had been determined. However, the contribution of sub-cortical mechanisms to multi-whisker integration should not be overlooked; an extended version of the model will be required to explore this important issue in more detail.

### Extending the model

Tactile stimuli which include three or more whiskers cause suppressive interactions across barrel cortex which serve to enhance the representation of complex multi-whisker deflection patterns [16], [19], [24], [61]. We investigated how additional whiskers are represented according to the model, by simulating the effect of a stimulus moving at various speeds through a row of whiskers which included two, three, four, or five whiskers (see Figure S3). When the whiskers were deflected simultaneously, the resulting activity across L2/3 was widespread and large and formed a symmetrical pattern, but when the whiskers were deflected consecutively the activity decreased across L2/3 in the direction corresponding to the stimulus motion. In agreement with previous studies the model predicts the existence of an activity gradient that is steeper for slower stimulus motions.

A previous modelling study suggested that a spatial gradient in the afferent activation of L2/3 could represent the direction of stimulus motion through the whisker field, and that this representation in L2/3 would be sharpened by recurrent inhibitory interactions [44]. The present model did not consider recurrent inhibition, which is prevalent in L2/3 [35], [62]–[64], because it considered primarily how subthreshold inputs interact to generate the earliest spikes in L2/3 (see Figure S1). We are currently working on a model which extends the present study and that of ref. [44], to test the hypothesis that regions of contrast in activity due to initial feed-forward interactions are enhanced by subsequent lateral inhibition. This model will also explore how stimulus coding might be affected by distance-dependent weights on synaptic connections, as suggested by recent experiments [62], [64].

### The impact of neural geometry on neural computation

The present simulation results afford an existence proof for a more general hypothesis that the geometry of projections between neighbouring cortical columns could be useful for encoding relative inter-sensor motion speed and direction.

In its weakest form the implication of the hypothesis is that interconnection geometry and connection speeds should be considered in detailed cortical microcircuit models if they are to accurately predict the response properties of individual cortical neurons. Given the remarkable spatial relationship between the whisker and its associated barrel column, it is surprising that, with the exception of refs. [65], [66] and our own previous model [44], connection geometry has not been an important factor in computational neuroscience models of the barrel system.

In its strongest form the implication is that the cortex could carry out specific computations by reading out the tangential position of active cortical neurons. This is essentially the same idea as the place theory proposed by Jeffress [1]. The principle behind our model and the Jeffress model are essentially the same. In both, a bank of coincidence detectors receive input from spatially separated sources after delays governed by the distance from either source, and thus activity in detectors whose connection delays compensate that of the stimulus motion reports the stimulus velocity. It remains to be shown whether tactile specialists such as rats and mice can discriminate adjacent whisker contact times over the range generated in the model, although emerging techniques are allowing the link between barrel cortex activity and performance on tactile discrimination tasks to be explored in unprecedented detail [67].

Jeffress’ place theory can be thought of as a specific case of a more powerful computational principle, recently termed ‘polychronous wavefront computation’ (PWC) [68]. In PWC terminology, two sources in the Jeffress model specify a one-dimensional axis through a medium (the axonal web), along which the placement of detector neurons determines their inter-stimulus interval selectivity. However, sources and detectors can be arranged in two- or higher- dimensional media, such as the barrel cortex, to perform non-trivial computations. The barrel cortex, with the precise correspondence between the grid of cortical columns and the grid of whisker sensors, is an ideal structure in which to investigate the role of neural geometry in neural computation.

The simplicity of the current model affords its explanatory power. However, a future study will be required to verify under what conditions the behaviour of the model is retained, when many hundreds of neurons and thousands of synaptic contacts are modelled explicitly. The barrel column is currently the target of a number of detailed modelling efforts [39], [69]–[71]. Complementing these approaches, the power of our simple geometric model to explain a series of complex observations suggests that the geometry of synaptic connections in and between barrel columns should be considered if we are to understand the function of cortical microcircuitry.

## Supporting Information

Figure S1
**Analysis of spike timing.** Spike histograms were constructed for neurons at different locations in 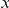 (shown in successive panels). In each panel, rows correspond to different inter-whisker deflection intervals (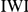), and columns show progressive simulation time. Each pixel shows the average spike count, across 5000 trials, in a 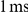 window. Histograms are aligned by 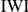 such that white ticks indicate the onset of the influence of whisker A (the first of which is labelled 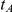 in the first panel), and grey ticks indicate the onset of the influence of whisker B (labelled 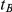). Specifically, ticks are at 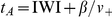 and 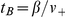, which is the time at which excitation from each whisker registers at the neuron closest to the corresponding input source (at 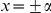). In general, neurons spiked at low rates, in time with the influence of the closer whisker (diagonal versus linear trends for 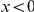 or 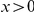 respectively). For neurons located around the midline additional spikes occurred in time with the second whisker deflection. Interestingly, in many cases additional spikes occurred in the millisecond before the influence of the second whisker, indicating a delayed influence of the first. The maximum average spike count was 0.82 spikes per stimulus at 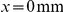 and 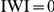, in the millisecond following the influence of whisker B.(TIFF)

Figure S2
**Constraints on the timing of axonal propagation.** The delay on the onset of inhibition, 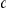, required to make excitation and inhibition from the same whisker arrive coincidently, is plotted for varying inhibitory connection speeds 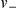 at three locations in L2/3. Solutions to the equation 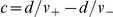 are plotted for three different L4 to L2/3 inter-soma distances: First to the home barrel center 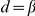 (solid line), second to the adjacent barrel center 
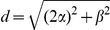
 (dashed line), and third to two barrel centers away 
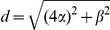
 (dotted line). All other parameters were fixed at the values reported in the main text (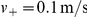, 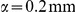, and 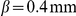). For choices of the parameters 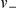 and 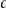 that are above a line, inhibition will arrive at L2/3 neurons above the corresponding barrel center later than excitation evoked by the same whisker, and vice versa for parameters that fall below that line. The cross indicates the choice of 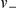 and 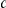 used for the simulations in the main text, which make excitation and inhibition coincident for neurons located approximately one barrel away from the source. Measurements of 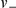 and 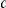 below the solid line would falsify the model because no facilitatory zone and hence no map for the inter-whisker interval could exist in L2/3. Values much greater than the dashed line would map inter-whisker intervals between adjacent barrel centers with poor coverage.(TIFF)

Figure S3
**Predicted responses to additional whiskers.** Responses across a large region of barrel cortex were generated by deflecting increasing numbers of whiskers. The top panel shows the mean spike count, over 5000 trials, to deflection of whisker A followed by whisker B after intervals ranging 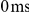 to 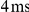 (see legend). Ticks along the 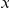
–axis mark the location of the barrel centers, at 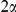 spacing, for columns corresponding to whiskers A to E in a row on the snout. The top panel is comparable with Figure 7 from the main text. Successive panels include deflections of additional whiskers, each deflected a fixed time after deflection of the adjacent whisker to the left. When three or more whiskers are deflected simultaneously (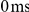 interval) the response resembles the superposition of adjacent two-whisker tuning functions, punctuated by additional peaks. When stimulated consecutively, the two-whisker tuning function between each pair of columns is modulated by an overall response decrease in the direction corresponding to the stimulus movement direction. Thus, when additional whiskers are included by tactile stimuli, the model predicts an overall trend for responses to decrease in the direction of the stimulus movement.(TIFF)
