# Supplementary figures and images for: Neural Computation via Neural Geometry: A Place Code for Inter-whisker Timing in the Barrel Cortex?
Source: PLoS Comput Biol. 2011 Oct 13;7(10):e1002188. doi: 10.1371/journal.pcbi.1002188 (PMC3192806; doi:10.1371/journal.pcbi.1002188)

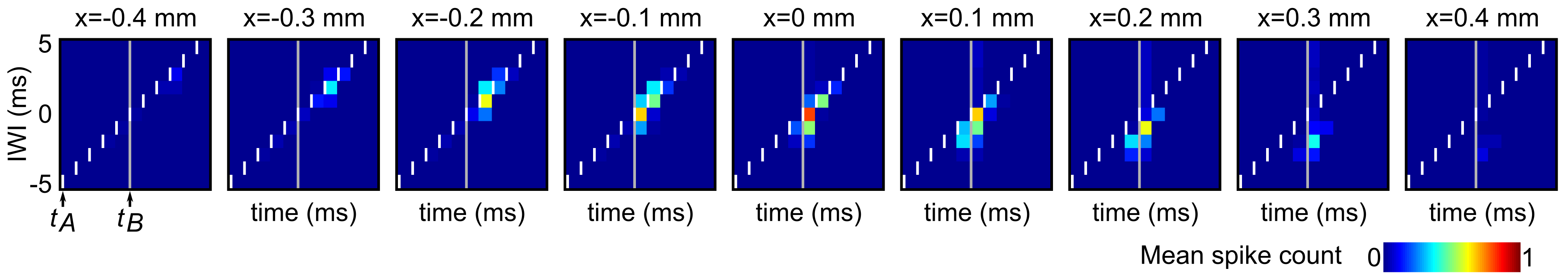

Supplement: Figure S1 — Analysis of spike timing. Spike histograms were constructed for neurons at different locations in (shown in successive panels). In each panel, rows correspond to different inter-whisker deflection intervals (), and columns show progressive simulation time. Each pixel shows the average spike count, across 5000 trials, in a window. Histograms are aligned by such that white ticks indicate the onset of the influence of whisker A (the first of which is labelled in the first panel), and grey ticks indicate the onset of the influence of whisker B (labelled ). Specifically, ticks are at and , which is the time at which excitation from each whisker registers at the neuron closest to the corresponding input source (at ). In general, neurons spiked at low rates, in time with the influence of the closer whisker (diagonal versus linear trends for or respectively). For neurons located around the midline additional spikes occurred in time with the second whisker deflection. Interestingly, in many cases additional spikes occurred in the millisecond before the influence of the second whisker, indicating a delayed influence of the first. The maximum average spike count was 0.82 spikes per stimulus at and , in the millisecond following the influence of whisker B. (TIFF) [file pcbi.1002188.s001.tiff]

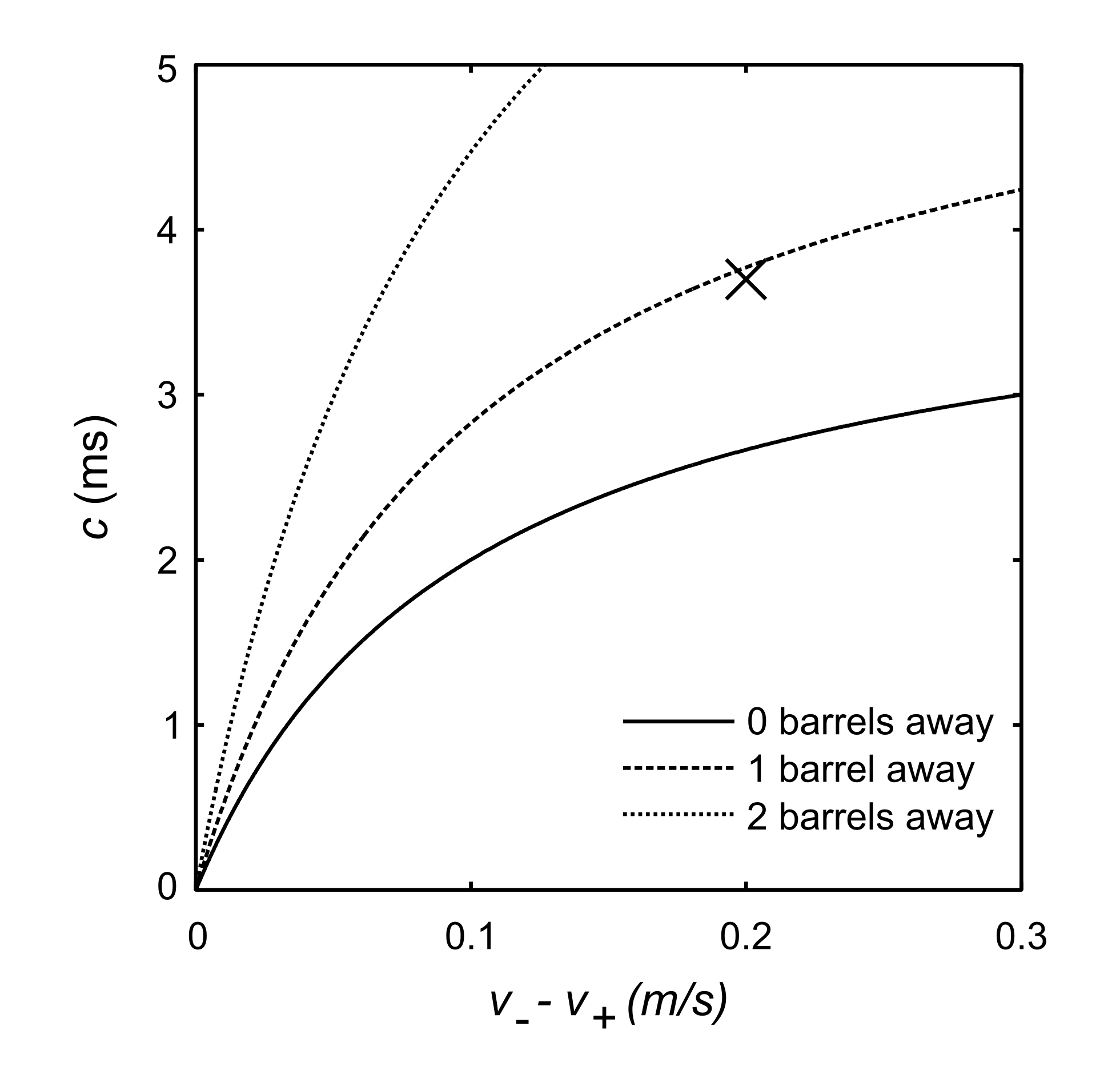

Supplement: Figure S2 — Constraints on the timing of axonal propagation. The delay on the onset of inhibition, , required to make excitation and inhibition from the same whisker arrive coincidently, is plotted for varying inhibitory connection speeds at three locations in L2/3. Solutions to the equation are plotted for three different L4 to L2/3 inter-soma distances: First to the home barrel center (solid line), second to the adjacent barrel center (dashed line), and third to two barrel centers away (dotted line). All other parameters were fixed at the values reported in the main text (, , and ). For choices of the parameters and that are above a line, inhibition will arrive at L2/3 neurons above the corresponding barrel center later than excitation evoked by the same whisker, and vice versa for parameters that fall below that line. The cross indicates the choice of and used for the simulations in the main text, which make excitation and inhibition coincident for neurons located approximately one barrel away from the source. Measurements of and below the solid line would falsify the model because no facilitatory zone and hence no map for the inter-whisker interval could exist in L2/3. Values much greater than the dashed line would map inter-whisker intervals between adjacent barrel centers with poor coverage. (TIFF) [file pcbi.1002188.s002.tiff]

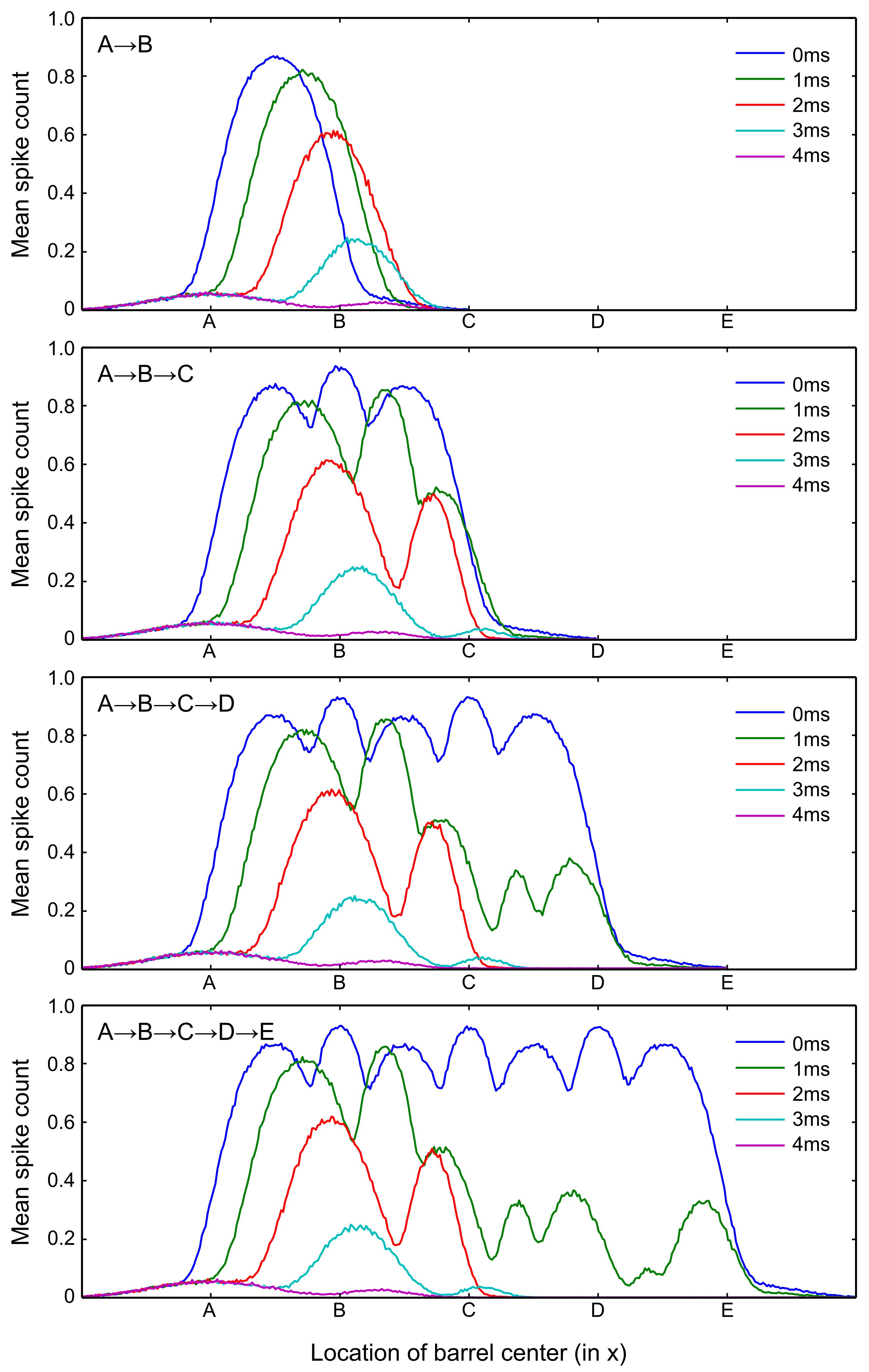

Supplement: Figure S3 — Predicted responses to additional whiskers. Responses across a large region of barrel cortex were generated by deflecting increasing numbers of whiskers. The top panel shows the mean spike count, over 5000 trials, to deflection of whisker A followed by whisker B after intervals ranging to (see legend). Ticks along the –axis mark the location of the barrel centers, at spacing, for columns corresponding to whiskers A to E in a row on the snout. The top panel is comparable with Figure 7 from the main text. Successive panels include deflections of additional whiskers, each deflected a fixed time after deflection of the adjacent whisker to the left. When three or more whiskers are deflected simultaneously ( interval) the response resembles the superposition of adjacent two-whisker tuning functions, punctuated by additional peaks. When stimulated consecutively, the two-whisker tuning function between each pair of columns is modulated by an overall response decrease in the direction corresponding to the stimulus movement direction. Thus, when additional whiskers are included by tactile stimuli, the model predicts an overall trend for responses to decrease in the direction of the stimulus movement. (TIFF) [file pcbi.1002188.s003.tiff]
